# The effects of population management on wild ungulates: A systematic map of evidence for UK species

**DOI:** 10.1371/journal.pone.0267385

**Published:** 2022-06-10

**Authors:** Owain Barton, Amy Gresham, John R. Healey, Line S. Cordes, Graeme Shannon

**Affiliations:** 1 School of Natural Sciences, Bangor University, Bangor, Gwynedd, United Kingdom; 2 School of Ocean Sciences, Bangor University, Menai Bridge, Anglesey, United Kingdom; Auburn University, UNITED STATES

## Abstract

**Introduction:**

Over recent decades, the abundance and geographic ranges of wild ungulate species have expanded in many parts of Europe, including the UK. Populations are managed to mitigate their ecological impacts using interventions, such as shooting, fencing and administering contraception. Predicting how target species will respond to interventions is critical for developing sustainable, effective and efficient management strategies. However, the quantity and quality of evidence of the effects of interventions on ungulate species is unclear. To address this, we systematically mapped research on the effects of population management on wild ungulate species resident in the UK.

**Methods:**

We searched four bibliographic databases, Google Scholar and nine organisational websites using search terms tested with a library of 30 relevant articles. Worldwide published peer-reviewed articles were considered, supplemented by ‘grey’ literature from UK-based sources. Three reviewers identified and screened articles for eligibility at title, abstract and full-text levels, based on predefined criteria. Data and metadata were extracted and summarised in a narrative synthesis supported by structured graphical matrices.

**Results:**

A total of 123 articles were included in the systematic map. Lethal interventions were better represented (85%, n = 105) than non-lethal interventions (25%, n = 25). Outcomes related to demography and behaviour were reported in 95% of articles (n = 117), whereas effects on health, physiology and morphology were studied in only 11% of articles (n = 14). Well-studied species included wild pigs (n = 58), red deer (n = 28) and roe deer (n = 23).

**Conclusions:**

Evidence for the effects of population management on wild ungulate species is growing but currently limited and unevenly distributed across intervention types, outcomes and species. Priorities for primary research include: species responses to non-lethal interventions, the side-effects of shooting and studies on sika deer and Chinese muntjac. Shooting is the only intervention for which sufficient evidence exists for systematic review or meta-analysis.

## Introduction

Wild ungulates are integral to the functioning of grassland and forest ecosystems [1–4]. As highly mobile and wide-ranging herbivore species, they have the capacity to influence ecological processes at multiple spatial scales [5–7]. In recent decades, the abundance and geographic ranges of many ungulate species have rapidly increased across Europe [8, 9]. Population growth has been attributed to translocations, the removal of natural predators, climate change and widespread alterations in land use [10]. These include the increased planting of trees to meet conservation targets, which has formed suitable habitat for a range of ungulates and agricultural intensification that provides a consistently available food-source throughout the year [11–13]. As their densities increase, a variety of interacting ecological and social factors must be considered in order to manage ungulate populations sustainably and satisfy the objectives of a range of stakeholders, including foresters, conservationists, farmers, landowners, recreational hunters and countryside visitors [10, 14, 15].

The effects of wild ungulates on ecosystems are species- and context-specific. Low-level herbivory by deer (Cervidae) and feral goats (*Capra* spp.) has been shown to suppress the growth of competitively dominant plant species and accelerate nitrogen and carbon cycling [6, 16]. However, more intense browsing pressure has been linked to declines in biodiversity [17], reductions in forest understorey foliage [18] and damage to agriculture [19]. In wetlands, rooting by wild pigs (*Sus scrofa*) can enhance microhabitat diversity and plant species richness [20], whereas the same behaviour in forests has been associated with decreased plant diversity [21] and the destruction of habitat for small mammals [22].

In human transformed landscapes, ungulates pose a threat to human health and well-being as a result of road traffic accidents [23, 24]. A recent assessment of the frequency of ungulate-vehicle collisions (UVCs) in Europe estimated that 30,000 incidents occur each year [25]. Additionally, ungulates are known to act as reservoirs of diseases, such as bovine tuberculosis [26] and salmonella [27], as well as vectors of diseases, such as Lyme disease [28], that are transmissible to humans and domestic livestock [29, 30]. In the past two decades, the need to better understand the role of ungulates as ecosystem engineers and to mitigate their negative ecological and socio-economic impacts has been increasingly recognised by scientists, wildlife managers and conservationists [8, 14].

Methods to mitigate ungulate impacts include control interventions, such as shooting, administering contraception, non-lethal deterrents and supplementary feeding [10, 31, 32]. Typically, the efficacy of each practice is measured by observing how key environmental variables respond or monitoring changes in the prevalence of disease [8, 10]. For example, in the UK the effectiveness of shooting deer is often estimated by observing the relationship between shooting effort and browsing damage to sensitive woodlands [8, 10, 33]. Monitoring environmental indicators of ecological change (IECs) is relatively inexpensive and provides convenient metrics for managers to compare the efficacy of different management strategies [8, 15, 34]. However, target species may respond to an intervention in a variety of ways that if not appropriately considered could lead to management strategies being ineffective or even counter-productive. For example, in the case of red deer (*Cervus elaphus*), shooting has been shown to reduce population densities and effectively mitigate the environmental impact of browsing [35]. However, there is evidence that shooting also has long-term effects on the morphology of red deer [36] and that the disturbance of shooting causes shifts in their home ranges, which may promote the spread of diseases [37]. Localised shooting of deer can lead to the development of source-sink dynamics in the population that neutralise efforts to reduce numbers at the scale of the landscape or region [33, 38]. Additionally, responses of target species may be taxon-specific, which is particularly important in scenarios where a single intervention is applied to manage multiple species. For instance, supplementary feeding can reduce levels of bark damage by red deer [39] but this intervention has also been shown to promote the population growth of wild pigs, leading to an increase in their disturbance on the environment [40].

A recent review [14] emphasised the importance of developing strategies for adaptive population management informed by robust empirical evidence. A total of ten measures were proposed to ensure the viability and long-term persistence of ungulate populations. These included long-term monitoring of habitat performance indicators (e.g., species richness), analysis of the indirect and unintended effects of supplementary feeding and a recognition for the impacts of hunting beyond reducing population densities [14]. Accurate assessment of the responses of ungulate species to interventions typically requires intensive sampling [e.g., 41] and specialist equipment, such as motion-activated cameras or global-positioning system (GPS) collars [e.g., 42]. These approaches are typically unfeasible for most practitioners and formal studies are usually constrained to observations of a narrow range of responses for a single species or intervention. Consequently, individual studies may be of limited benefit to decision-makers faced with the challenge of developing strategies to manage multiple species simultaneously in order to meet a range of objectives (e.g., environmental impact mitigation, sustainable exploitation, reducing disease transmission). Therefore, syntheses of the literature, that provide information on the quantity and quality of the available evidence are needed to provide appropriate support for wildlife and land managers as well as policymakers. However, systematic assessments of the available evidence are lacking. This is of particular importance for wild ungulate management because the strength of the evidence-base supporting practices is unclear.

In this review we systematically map the evidence for the effects of control interventions on the wild ungulate species resident in the UK. The purpose of the systematic map is to collate, catalogue and describe the extent and distribution of evidence in relation to key variables [e.g., species, intervention type, response etc., 43]. Additionally, the map is used to identify important topics for primary research and serves as a valuable resource for scholars to more easily locate relevant articles for further systematic review or meta-analyses. The aim of the study is to support the development of more efficient and effective management strategies by collecting and characterising the evidence for species responses to commonly-adopted practices.

### Scope of study

The primary objective of this systematic map is to collate existing research on the effects of management practices on the nine wild ungulate (Artiodactyla) species resident in the UK. Searches were restricted to these species to provide an appropriate focus and to ensure that the volume of literature screened for eligibility would be manageable. The species included represent a range of body sizes and ecological characteristics (e.g., feeding behaviour, reproduction rates, average lifespan etc.). Several (notably wild pigs, red deer and roe deer) are also abundant across Europe and are globally important for wildlife management [8, 44]. Worldwide searches were conducted for peer-reviewed research articles but searches for ‘grey’ literature were restricted to UK-based sources only. It was beyond the scope of this review to critically appraise the evidence collected for each species. Instead, the synthesis provides a species-specific summary of the available evidence to identify important knowledge gaps and prioritise topics for future research and/or evidence synthesis. We did not preregister a protocol for this systematic map. In all other respects our procedure followed guidelines established by the Collaboration for Environmental Evidence [45] and complies with PRISMA and ROSES reporting standards [46, 47, S1 and S2 Files]

### Primary question

What evidence is available on the effects of control interventions, such as fencing, shooting, administering contraception, supplementary feeding and non-lethal deterrents, on the wild ungulate species that are resident in the UK?

## Methods

### Eligibility criteria

Eligible articles included any primary research study that collected data by way of an experiment or quasi-experiment (control-intervention and/or before-after) to examine the effects of an intervention on one or several features of ungulate biology. Articles originating from any country were considered for inclusion. No explicit date restrictions were applied but the date of the earliest available records varied between literature sources. Articles were required to meet the eligibility criteria for the elements of the primary question described in the following sections.

#### Population

All wild ungulate (Artiodactyla) species and subspecies currently resident in the UK, as described by Apollonio, Andersen and Putman [8] including:

**Table:** 

Chinese muntjac	*Muntiacus reevesi*
Chinese water deer	*Hydropotes inermis*
Fallow deer	*Dama dama*
Feral goats	*Capra aegagrus hircus*
Feral sheep	*Ovis aries*
Red deer (accepted sub-species common name: Scottish red deer)	*Cervus elaphus* (accepted sub-species: *elaphus* or *scoticus*)
Roe deer	*Capreolus capreolus*
Sika deer	*Cervus nippon*
Wild pigs*	*Sus scrofa*

* Following the advice of Keiter et al. [48], the term ’wild pigs’ is used as the common name for *Sus scrofa*, which may be described in articles by a range of common names including wild boar, feral pigs and feral hogs.

NOTE: If the population was a sub-species described by a scientific or common name that is not resident in the UK (e.g., *Sus scrofa sibiricus* or elk), the article was excluded. If no sub-species was named and no common name was used, (e.g., *Sus scrofa* or *Cervus elaphus*) the article was included.

#### Interventions

Deliberate human practices intended to mitigate the environmental and socio-economic impacts of wild ungulates by manipulating one or more features of their biology. Included in the review are interventions that directly influence target species such as shooting, administering contraception, supplementary feeding and non-lethal deterrents, as well as actions that have indirect effects, such as fencing and landscape modification. All practices considered are hereafter referred to as ‘interventions’.

#### Comparator

No intervention (a separate control site or population in a control/intervention (CI) study design, a time period of no intervention in a before/after (BA) study design or a combination of both in a before/after/control/intervention study design (BACI)) or an alternative level of intervention intensity (e.g., the effect of shooting may be inferred by comparing sites, populations or time periods that experienced different levels of shooting effort in an observational (Obs) study).

#### Outcomes

Any responses of the target species to interventions were reported as they were stated in the relevant articles. Any effects on the biology of the target species were considered, including influences on population size and viability, morphology, physiology, movement behaviour, life history traits and habitat selection. The only outcomes included were effects on the target species and not secondary effects on other species, disease prevalence, plant and animal communities or habitat ecosystem components (e.g., evidence of the influence of an intervention on habitat selection by individuals of the target species from GPS location data or pellet counts was included as an outcome, but not inference from variation in tree growth or local species richness).

### Searching for articles

An initial scoping search was conducted to identify suitable search terms, estimate the volume of relevant literature and validate the search methodology. Details of the search terms, number of hits and comments on the general quality of identified articles were recorded (S3 File). Terms describing the populations of interest were linked to intervention terms to form the following search string that was used to query Internet search engines and online bibliographic databases:

*Population*: ts = (muntjac OR "muntiacus reevesi" OR "chinese water deer" OR "hydropotes inermis" OR "roe deer" OR "capreolus capreolus" OR "red deer" OR "cervus elaphus" OR "sika deer" OR "cervus nippon" OR "fallow deer" OR "dama dama" OR "feral goat*" OR "capra aegagrus hircus " OR "wild goat*" OR "feral pig" OR "sus scrofa" OR "feral pigs" OR "feral hog*" OR "feral swine" OR "wild pig" OR "wild pigs" OR "wild hog*" OR "wild boar" OR "feral sheep")

AND

*Intervention*: ts = ("population control" OR "lethal control" OR hunt* OR cull* OR shoot* OR harvest* OR stalk* OR bait* OR poison* OR trapping OR (inhibit* AND reproduc*) OR immunocontracept* OR contracept* OR "fertility control" OR repel* OR deterrent* OR "diversionary feed*" OR (supplement* AND feed*) OR (supplement* AND food) OR "feed* station$" OR "forest management" OR "landscape structure" OR (manipulat* AND landscape) OR (manipulat* AND habitat) OR fenc*)

### Sources of publications

A range of online sources were searched including four bibliographic databases (Clarivate Analytics Web of Science Core Collection and BIOSIS Citation Index, CAB Direct, Open Grey (www.opengrey.eu) and EThOS (www.ethos.bl.uk)), nine organisational websites and Google Scholar (S3 File). Where possible, search histories were saved in order to re-run the search if necessary. For each literature source, data were collected on: date accessed, search terms used, number of hits and a qualitative estimate of the relevance of identified articles (S3 File). Resource limitations constrained this study to an assessment of articles published in the English language.

### Article screening and data coding

Articles identified by the search string were screened for eligibility using the online open-source platform of CADIMA (www.cadima.info/index.php). The CADIMA platform compiles records into a single reference library, automatically removes duplicates and facilitates the screening of articles at three levels; (1) Title, (2) Abstract and (3) Full text. The number of results from each literature source was recorded. A team of three reviewers screened articles for eligibility and reviewer consistency was checked at each stage (S3 File). Data were extracted for all articles that met the inclusion criteria and coded in an Excel spreadsheet to record the following information:

- Author(s)
- Study date
- Title
- Publication title
- Publication type (Journal article, report, thesis etc.)
- Country/countries of origin
- Total study area (km^2^)
- Study duration (years)
- Study species
- Species status (native or non-native)
- Intervention(s)
- Outcome(s)
- Response data type
- Study design (BA, CI, BACI or Obs)

Species status (native or non-native) was based on the species ranges described by the IUCN Red List of Threatened Species (www.iucnredlist.org) and CABI Invasive Species Compendium (www.cabi.org/ISC). As escaped domestic animals, feral sheep and feral goats were considered to be non-native irrespective of country.

## Results

### Number and types of articles

**Fig 1:**
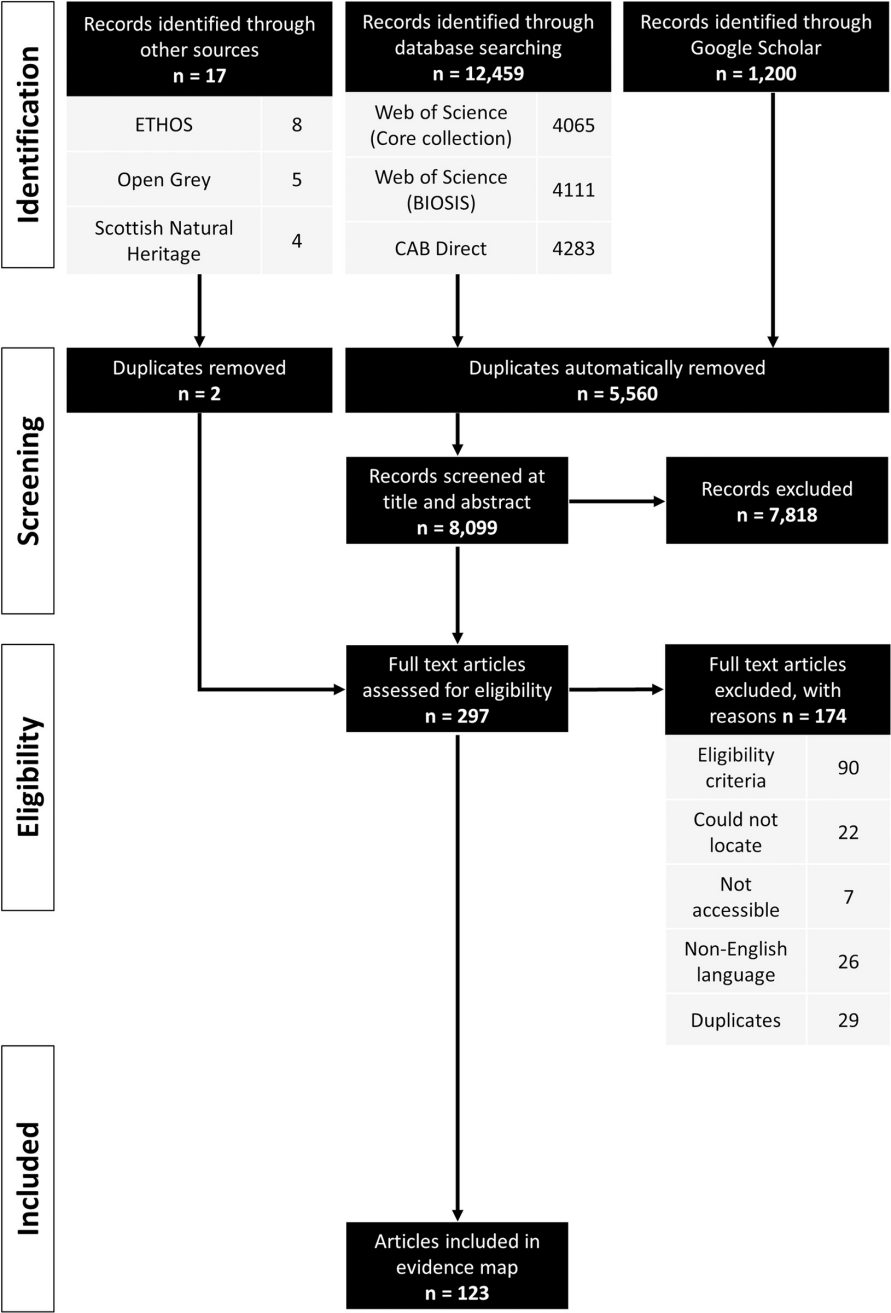
Flow diagram illustrating the number of articles gathered from each literature source, articles removed at each stage of screening and articles included in the evidence synthesis (diagram stages adapted from PRISMA guidance [47]).

Fig 1 illustrates the results of the literature searches and stages of article screening. A total of 13,659 articles were retrieved from bibliographic databases and Google Scholar, of which 5,560 were identified as duplicates and automatically removed by the CADIMA software. Very few articles (n = 17) were obtained from ’grey’ literature sources. Only 3% of articles (n = 297) were retained after screening at the title and abstract level. The list of articles was further reduced following full-text assessment to a subset of 123 articles that were used for data extraction. Of the articles excluded at the full-text assessment stage (n = 174), 52% were excluded because they did not meet the eligibility criteria (n = 90) and the remaining 48% were either not accessible (n = 7), not in the English language (n = 26), could not be located (n = 22) or were identified as duplicates (n = 29, S4 File).

**Fig 2:**
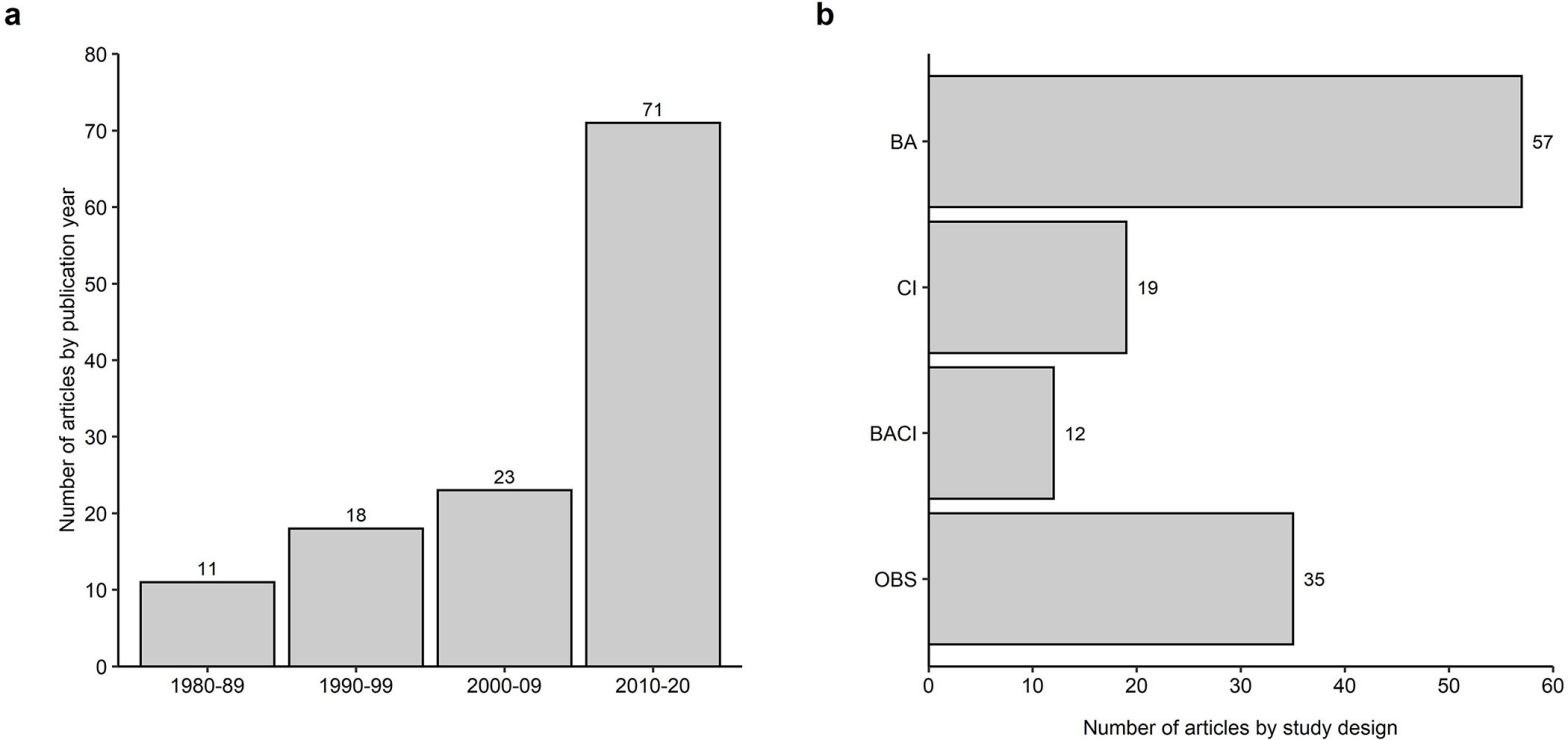
Number of articles by (**a**) publication year and (**b**) study design (BA = before-after, CI = control-intervention, BACI = before-after-control-intervention, and Obs = observation only). Totals are indicated by numerical values.

The duration of data-collection reported in articles ranged from less than 1 year to 27 years, excluding three studies that used multiple datasets [36, 49, 50]. The median duration of data collection was 3 years. Around 16% of articles used data collected over 10 or more years (n = 20). The earliest article included in the systematic map was published in 1980 (Fig 2A). We found a noticeable increase in the number of articles published in the past decade (2010 to 2020, n = 71), compared with the previous three decades (1980 to 2009, n = 52; Fig 2A). Studies designed to examine causal effects before and after an intervention were the most common and comprised 46% of the articles assessed (n = 57). Observational studies that quantified effects by observing sites or time periods exposed to different levels of intervention intensity accounted for around 28% of articles (n = 35). Approximately 15% of studies used designated control (non-treatment) and intervention (treatment) groups or sites (n = 19) and around 10% used a combined before-after-control-intervention study design (n = 12).

### Geographical representativeness and coverage of articles

**Fig 3:**
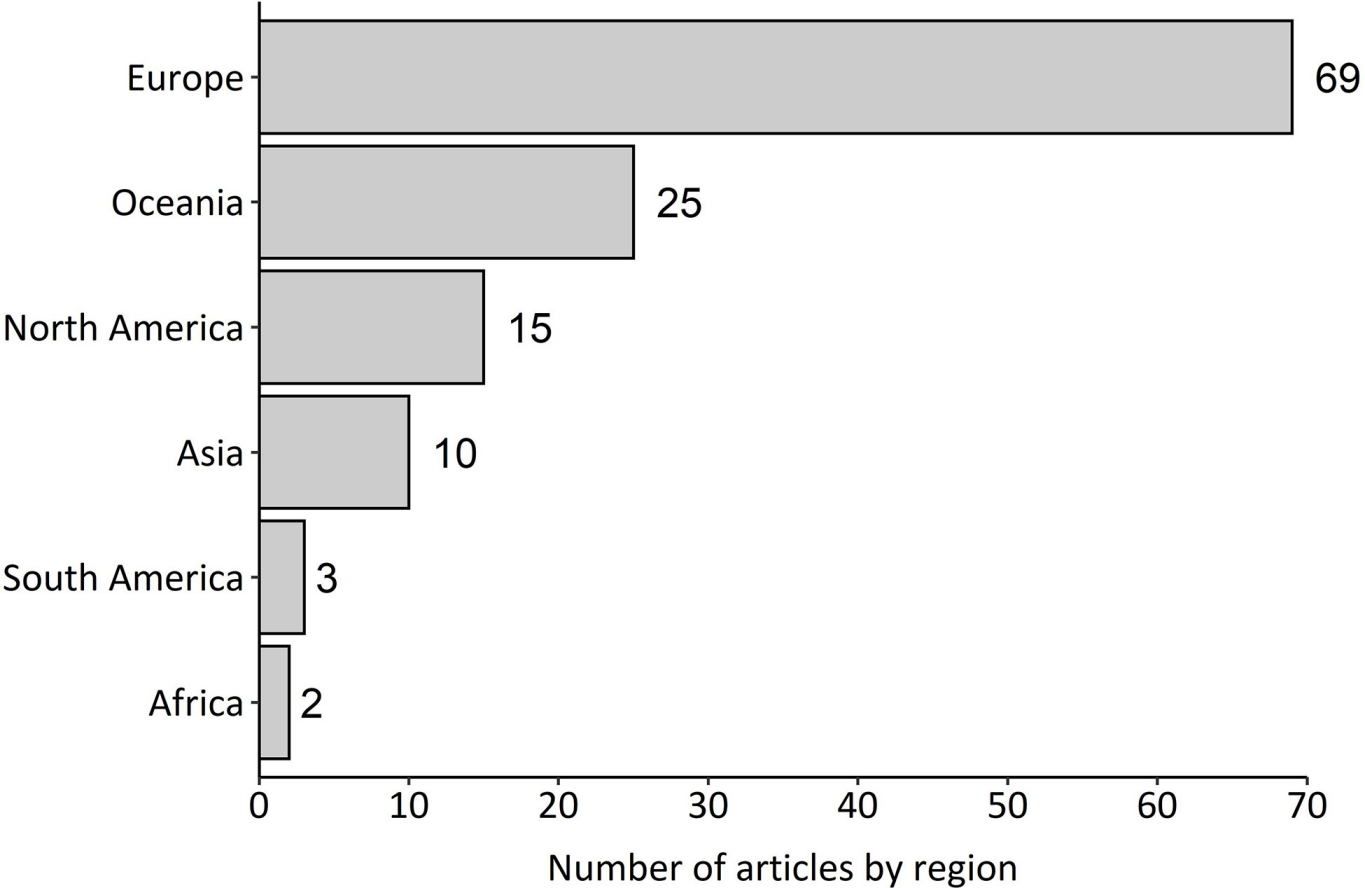
Number of articles by geographic region. Totals are indicated by numerical values.

**Fig 4:**
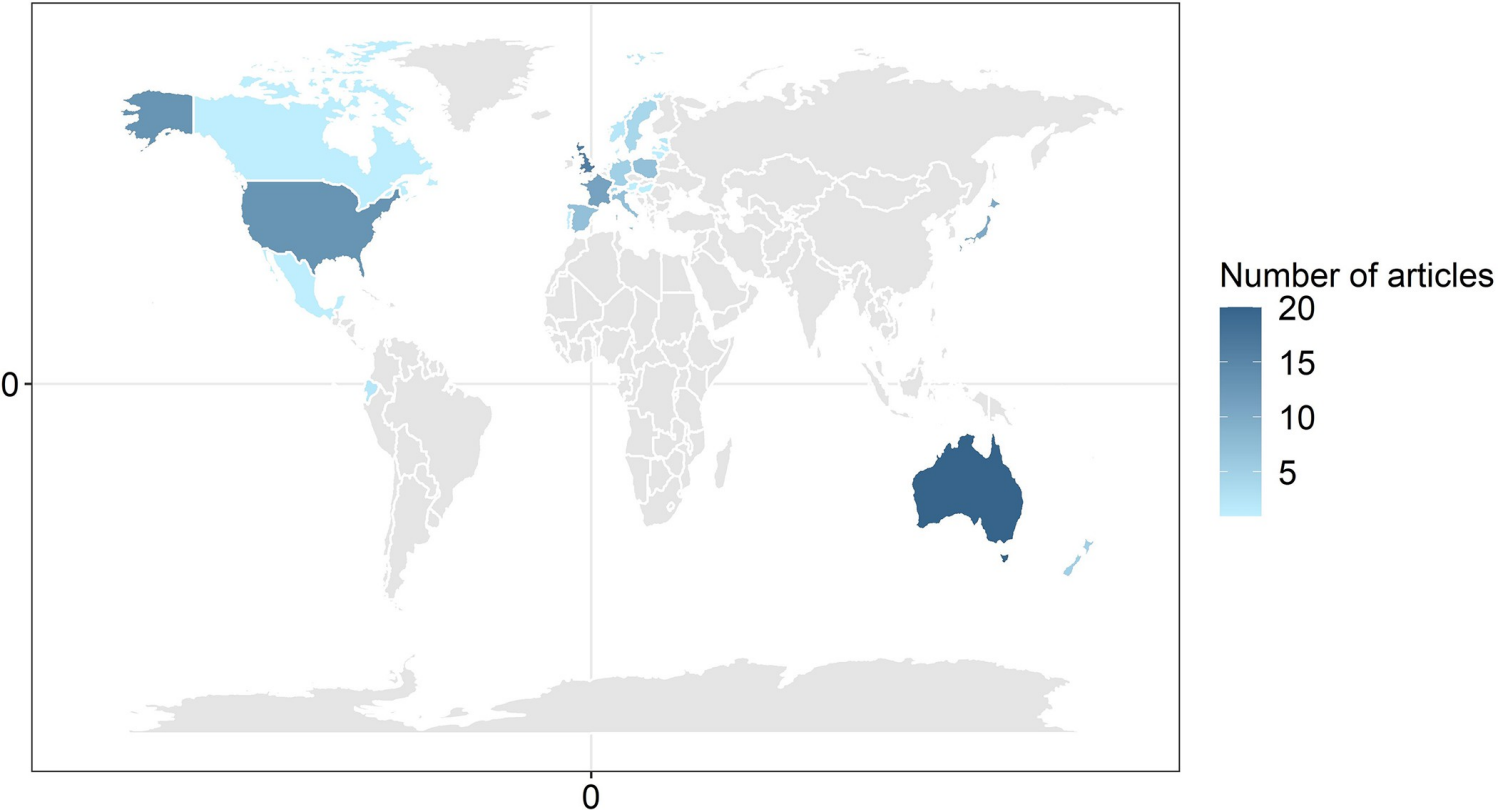
Geographical distribution of articles by country. Colours indicate the frequency of article occurrences. The map was developed using the ’ggplot2’ and ’maps’ packages in R (www.R-project.org), which utilise public domain data from Natural Earth (www.naturalearthdata.com).

**Fig 5:**
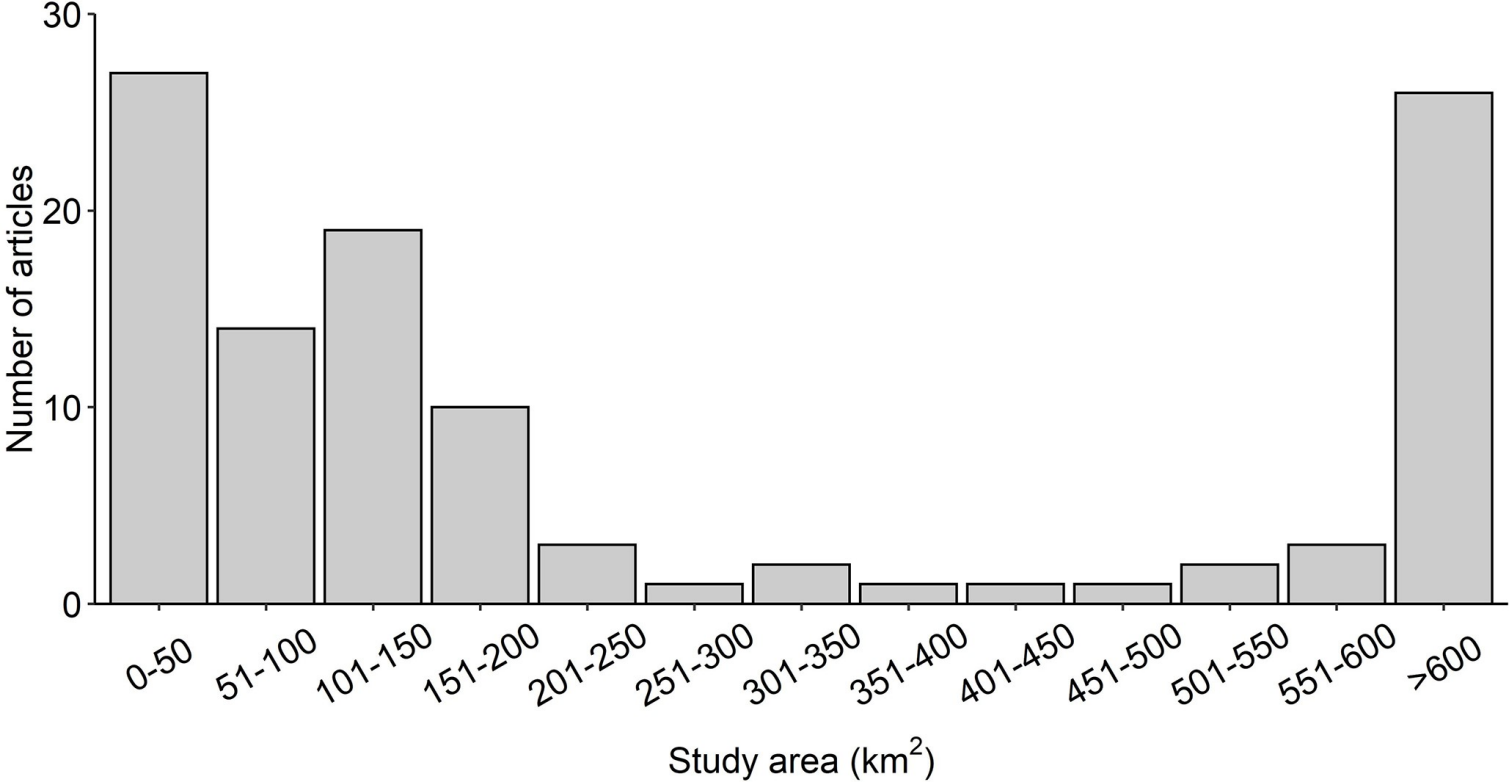
Number of articles by total study area (km^2^).

The geographic location of the studies reported in the articles included six regions (Fig 3). Europe was the most well-studied region with 69 articles. Oceania, North America and Asia were moderately well-studied with 25, 15 and 10 articles, respectively, while South America and Africa were the least-well studied regions with five articles between them. (Fig 3). The dataset used in the systematic map included articles from 28 countries (Fig 4). The most well-studied countries were Australia (n = 20), the UK (n = 16), USA (n = 13), France (n = 11) and Japan (n = 10, Fig 4). Study areas that covered more than one country were reported for five articles. The total area of land covered in each study ranged from less than 1 km^2^ to 175,000 km^2^. Articles most commonly covered study areas that were either 0–50 km^2^ or >600 km^2^ (Fig 5).

### Species representativeness

**Fig 6:**
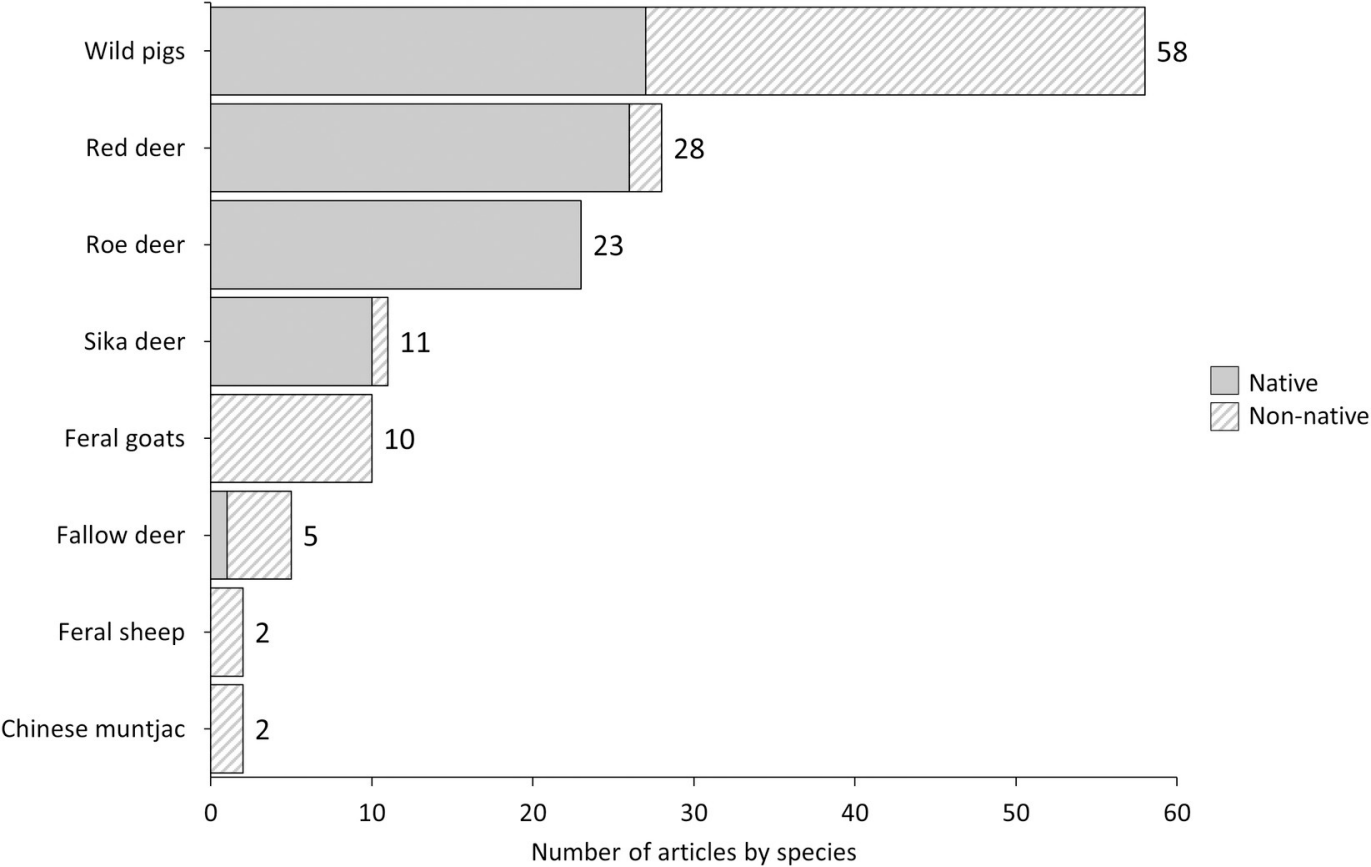
Number of articles for each of the ungulate species resident in the UK. Totals are indicated by numerical values. Patterns indicate the status of species studied in each article (native or non-native in relation to the geographic location of the study).

The number of articles included in the systematic map for each of the ungulate species resident in the UK is presented (Fig 6, no relevant articles were identified for Chinese water deer). Multiple species were reported in 13 articles. Species were studied inside their native ranges in approximately 59% (n = 73) of articles and outside their native ranges in approximately 37% (n = 46) of articles. Around 3% (n = 4) of articles reported on multiple species, of which some were inside their native range and others were outside their native range. Wild pigs were the most well-studied species (n = 58), followed by red deer (n = 28) and roe deer (n = 23), whereas few studies reported on sika deer (n = 11), feral goats (n = 10), fallow deer (n = 5), feral sheep (n = 2) or Chinese muntjac (n = 2, Fig 6). Roughly equal numbers of articles reported on wild pigs inside (47%, n = 27) and outside (53%, n = 31) their native range. Articles that reported on roe deer (n = 23) and most of the articles that reported on red deer (93%, n = 26) and sika deer (91%, n = 10) were conducted inside their native ranges, while articles that reported on feral goats (n = 10), feral sheep (n = 2) and Chinese muntjac (n = 2), as well as the majority of articles for fallow deer (80%, n = 4), were conducted outside their native ranges.

### Types of interventions

**Fig 7:**
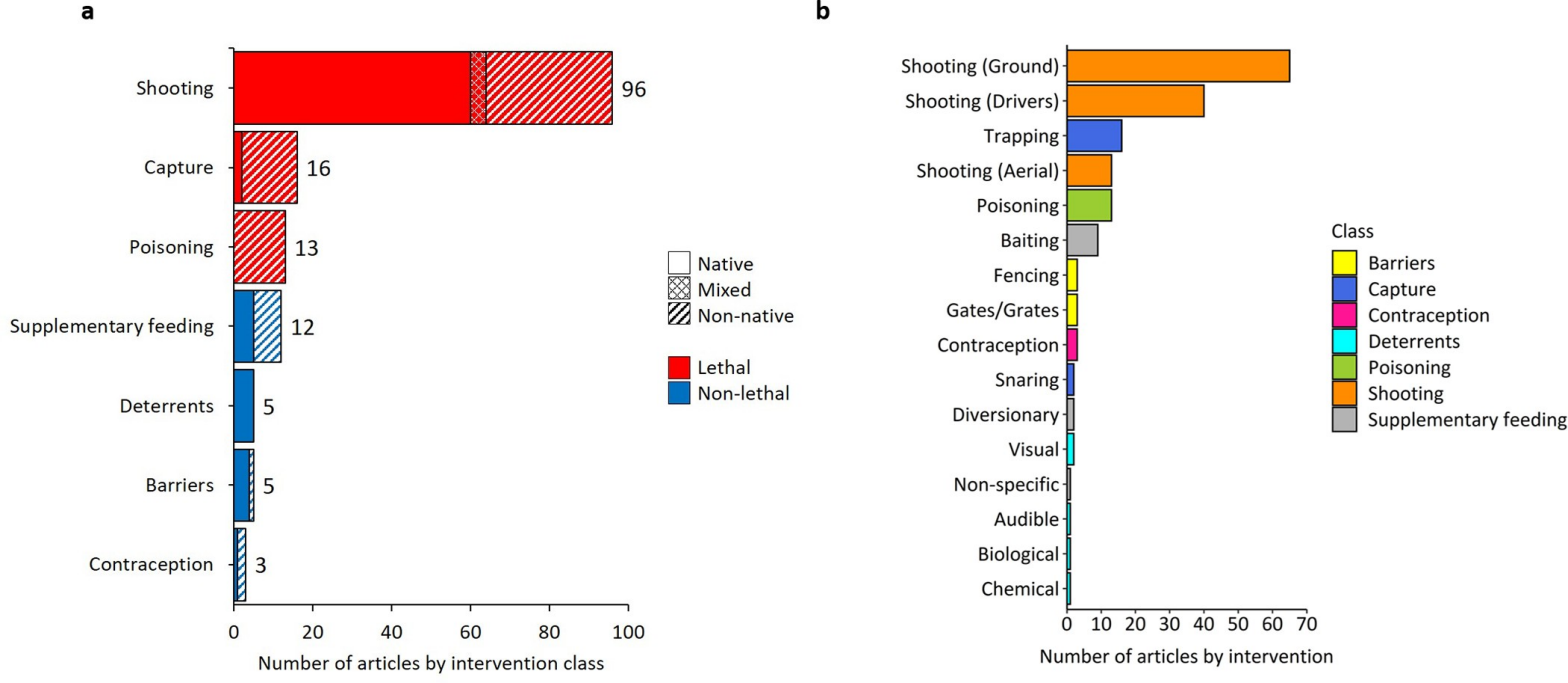
Number of articles by (**a**) intervention class and (**b**) intervention category. Totals are indicated by numerical values. Patterns indicate the status of species studied in each article (native, non-native or a mixture of native and non-native species), in relation to the geographic location of the study.

Interventions were categorised and grouped into seven broader classes (Table 1). Multiple interventions were reported in 34 of the 123 included articles. Fig 7A presents the extent and distribution of articles in each intervention class. Shooting was the most well-studied intervention class and was examined in 78% of included articles (n = 96). The top three most frequently documented intervention classes (shooting, capture and poisoning) involved lethal interventions (Fig 7A). Supplementary feeding was the most well-studied non-lethal intervention class but was examined in less than 10% of articles (n = 12). The distribution of articles for native versus non-native species was roughly equal for shooting, supplementary feeding and contraception (Fig 7A). Articles that reported on the effects of poisoning (n = 13) and the majority of articles that reported on the effects of capture (88%, n = 14), focussed on non-native species. Whereas, articles that reported on the effects of deterrents (n = 5) and most of the articles that reported on the effects of barriers (80%, n = 4), examined native species. The most frequently documented interventions were ground-based shooting (n = 65), shooting with the assistance of dogs or human drivers (battues, n = 40), trapping (n = 16), shooting from an aerial vehicle (n = 13) and poisoning (n = 13, Fig 7B). The seven interventions that comprise the classes of barriers, contraception and deterrents (Table 1) were each reported in fewer than five articles (Fig 7B).

**Table 1:** Types of interventions used in the articles included in the systematic map. Interventions are categorised and assigned to a broad intervention class.

Class	Category	Description
Barriers	Fencing	Man-made continuous barriers
	Gates/Grates	Man-made barriers for entrance/exit points
Capture	Trapping	Whole-animal capture
	Snaring	Part-animal capture
Contraception	Contraception	Administering contraception
Shooting	Shooting (Ground)	Shooting with a gun only
	Shooting (Aerial)	Shooting from an aerial vehicle
	Shooting (Drivers)	Shooting with the assistance of dogs or human drivers (battues), includes mustering
Deterrents	Audible	e.g., playback devices or bird-scarers
	Biological	e.g., grazing livestock
	Chemical	e.g., predator scents
	Visual	e.g., reflectors or lights
Poisoning	Poisoning	Use of lethal poison
Supplementary feeding	Baiting	Provision of food to assist shooting, capture or poisoning
	Diversionary	Provision of food to divert animals away from a site or vulnerable site component (e.g., crop trees)
	Non-specific	Provision of food without explicit reasoning of purpose other than to support population management*

*Studies that examined the effects of supplementary feeding used to increase survival or population growth to support recreational hunting were not included in the systematic map.

### Types of outcome

**Fig 8:**
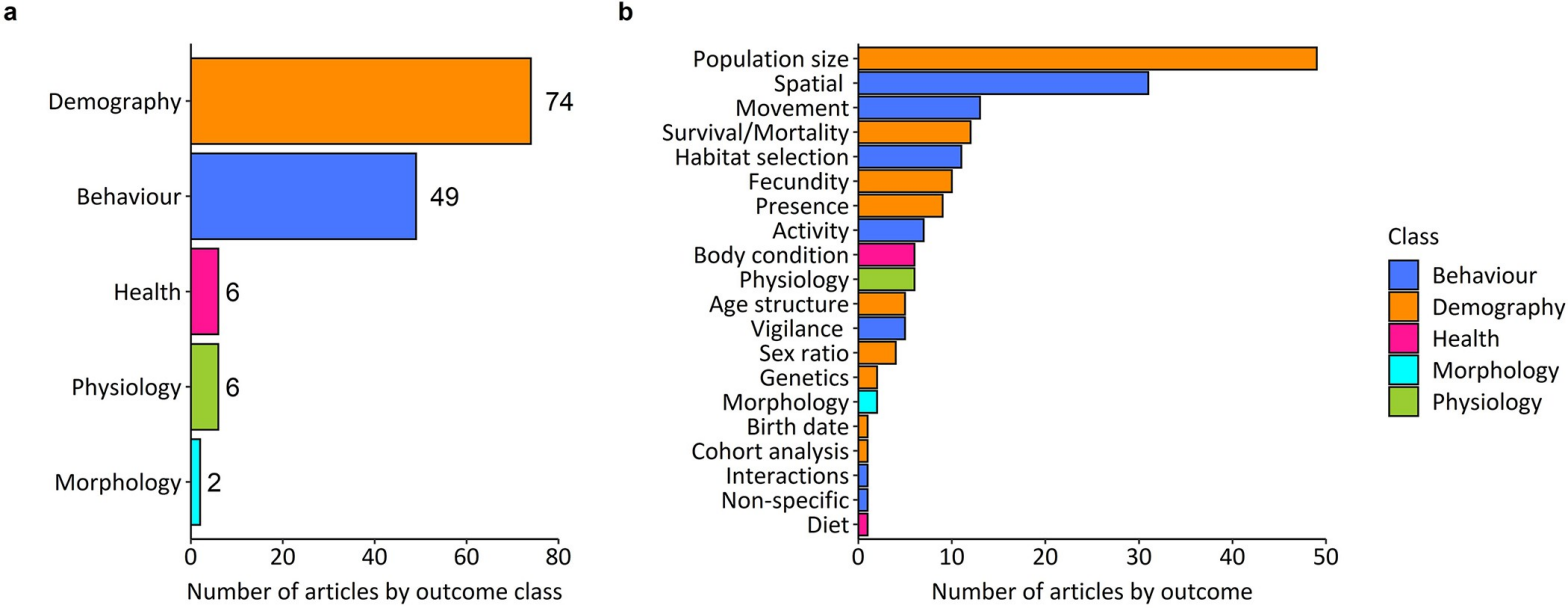
Number of articles by (**a**) outcome class and (**b**) outcome category. Totals are indicated by numerical values.

The outcomes of interventions were categorised and grouped into five broad classes (Table 2). Multiple outcomes were reported in 39 of the 123 included articles. Fig 8A presents the number of articles for each outcome class. Demography and behaviour were the most well-studied outcome classes and were examined in 60% (n = 74) and 40% (n = 49) of included articles, respectively. Health, morphology and physiology were each reported in fewer than 5% of articles (Fig 8A). The most frequently documented outcomes were effects on population size (n = 49), spatial behaviour (n = 31), movement behaviour (n = 13), survival or mortality (n = 12) and habitat selection (n = 11, Fig 8B).

**Table 2:** Types of outcomes reported in the articles included in the systematic map. Outcomes are categorised and assigned to a broad outcome class.

Class	Category	Description
Behaviour	Activity	Activity patterns over time (not spatially-explicit)
	Habitat selection	Space-use with explicit selection of sites or habitat types
	Interactions	Intra- or inter-specific interactions
	Movement	Movement distances, speeds and rates
	Non-specific	E.g., mating, grazing and sitting
	Spatial	Space-use (includes home range sizes, migrations, seasonal movements, distributions etc.)
	Vigilance	E.g., head-up movements
Demography	Age structure	Proportions of individuals per age class
	Birth date	Timing/date of birth
	Cohort analysis	Proportion of individuals of each sex in age classes
	Fecundity	Reproductive output or potential (includes litter size, number of corpora lutea, reproductive success, proportion of pregnant females etc.)
	Genetics	Population genetics
	Population size	Density or abundance
	Presence	Presence or absence
	Sex ratio	Proportions of each sex
	Survival/mortality	Proportion of population or sub-population surviving or dying between time periods
Health	Body condition	Weight, body fat levels, general condition
	Diet	Food types or species consumed
Morphology	Morphology	E.g., shape and size of antlers
Physiology	Physiology	E.g., level of stress hormones

### Linkages between interventions and outcomes

**Fig 9:**
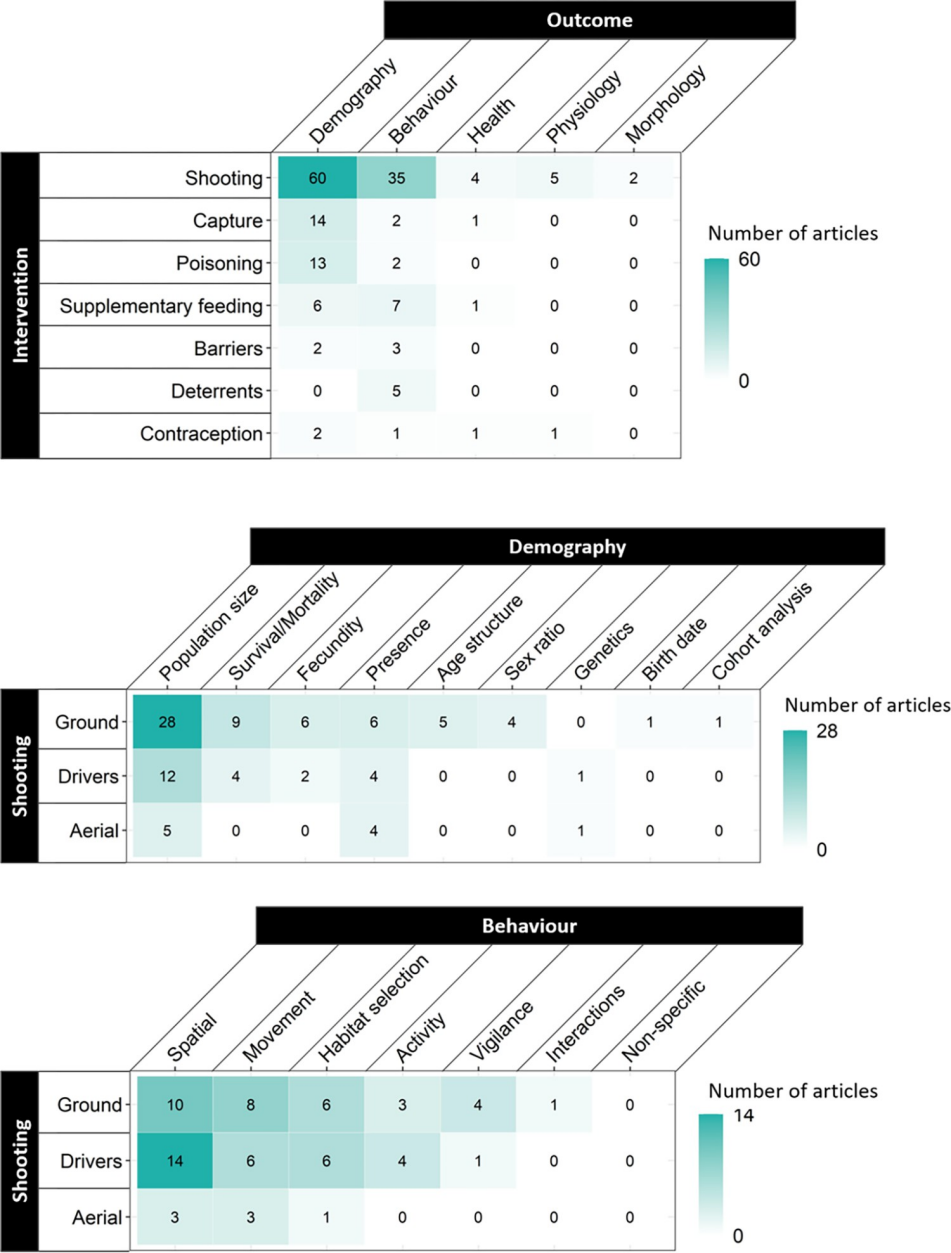
Structural matrices of the distribution and frequency of occurrence of studies reporting on the linkages between (**a**) intervention classes and outcome classes (**b**) shooting intervention categories and demography outcome categories and (**c**) shooting intervention categories and behaviour outcome categories for ungulate species resident in the UK. Matrix structure is adapted from McKinnon et al. [51].

Fig 9A displays the number of articles linking the interventions and outcomes (both grouped by class, Tables 1 and 2) identified in our systematic map. Well-studied linkages may be suitable areas of focus for more in-depth review and critical evaluation. Poorly studied linkages that are relevant to population management or policy and decision-making may be promising areas for further research or investigation by practitioners. Of the 35 possible linkages between interventions and outcomes, 15 were not identified in any article and a further 16 were reported in fewer than ten articles (Fig 9A). The most well-studied linkages were those of shooting and demography (n = 60), shooting and behaviour (n = 35), capture and demography (n = 14), and poisoning and demography (n = 13). The distribution of articles within each intervention and outcome class is presented for linkages between shooting and demography (Fig 9B) and shooting and behaviour (Fig 9C). Within the demography class the most frequently reported linkages were between population size and ground-based shooting (n = 28) or shooting with the assistance of drivers (n = 12, Fig 9B). No studies linking ground-based shooting with population genetics were identified. Within the behaviour class there was a more even distribution of articles amongst linkages (Fig 9C). The most frequently reported linkages were between spatial behaviour and ground-based shooting (n = 10) or shooting with the assistance of drivers (n = 14). The linkages between these interventions and habitat selection and movement were reported in 6 to 8 articles each (Fig 9C).

**Fig 10:**
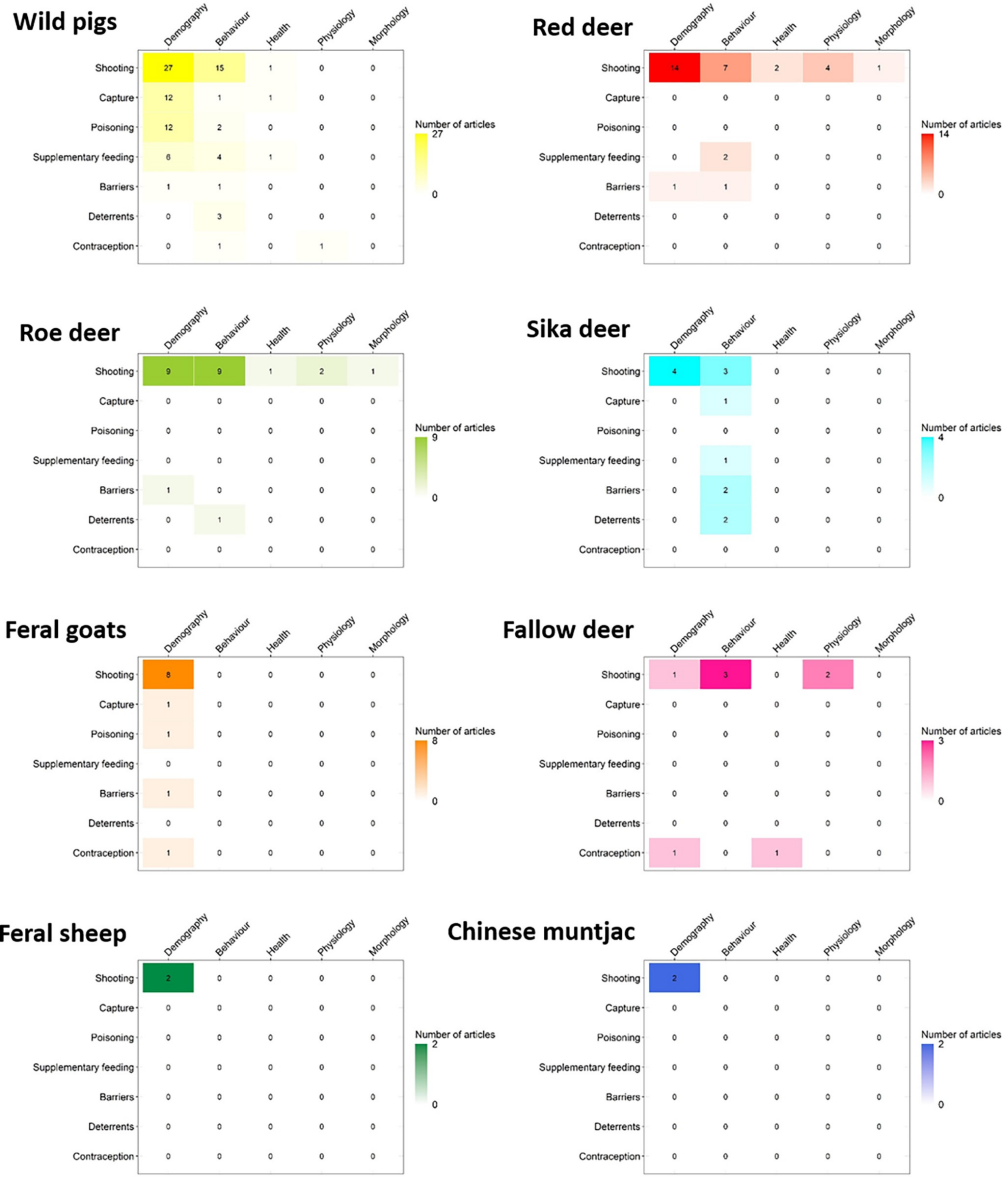
Structural matrices illustrating the distribution and frequency of articles on linkages between intervention classes and outcome classes for each ungulate species resident in the UK.

Fig 10 maps the intersection of invention classes and outcome classes for each species. Shooting was the only intervention to be investigated across all eight reported species (Fig 10). Articles that examined species responses to contraception were identified for wild pigs, feral goats and fallow deer only. Evidence for the effects of deterrents was limited to studies of wild pigs, roe deer and sika deer, and the effects of poisoning were restricted to wild pigs and feral goats. For red deer and roe deer evidence was almost exclusively related to shooting. Wild pigs were the only species for which evidence was available on their responses to all seven of the intervention classes. Most of the articles found for sika deer examined behavioural responses. For feral goats, feral sheep and Chinese muntjac evidence was limited to the effects of interventions on demography only (Fig 10).

## Discussion

Our review involved systematically mapping the existing worldwide research on the effects of population management interventions on the nine wild ungulate species that are resident in the UK. We collated peer-reviewed literature from 20 countries, supplemented by ’grey’ literature from UK-based sources to provide a species-specific summary of the evidence for commonly used interventions. The resulting map (S5 File) provides a resource for scholars, practitioners and decision-makers to more easily locate relevant articles, identify knowledge gaps and critically assess the state of the field.

### General comments

The results from our review describe important characteristics of the evidence-base and reveal significant unevenness in the distribution of research across species, interventions and the types of outcomes examined. The literature search identified 123 relevant articles after screening for eligibility. There was an upward trend in papers published over time. More articles have been published in the last decade (2010 to 2020, n = 71) than in the preceding three decades (1980 to 2009, n = 52). Overall, the robustness of the evidence-base is low and dominated by comparatively short-term studies that collected data for a median duration of three years. Long-term studies using data collected over 10 or more years were rare and accounted for only 16% of articles (n = 20). The majority of studies were conducted over large areas > 50 km^2^ (n = 96), 27% of these involved areas > 600 km^2^. Most of the articles originated from Europe, Oceania and North America, which is consistent with the geographic ranges of the species examined [9, 52, 53].

### Evidence extent

The relatively small number of articles included in our map (n = 123) most likely reflects a general trend towards studies evaluating the efficacy of management using only environmental-based indicators of ecological change [IEC, 15, 54]. A species is typically managed when its populations are negatively affecting human wellbeing, other species or ecosystem function [15]. Consequently, the outcomes reported by research are often environmental indicators, such as the frequency of ungulate-vehicle collisions, parasite loads and the growth rate/recruitment of plant species, and as such it often does not report metrics of the population of the wild ungulate species targeted by the intervention [14, 54]. Additionally, efforts to quantify ungulate species responses are constrained by limited human and financial resources. The notable scarcity of robust, long-term studies is likely due to the expense and logistical challenges associated with monitoring ungulate populations at the appropriate scale of the landscape or region [55, 56]. Technical advances and the decreasing cost of remote sensing technologies, such as motion-activated cameras and unmanned aerial vehicles, provide new opportunities for ungulate population monitoring, which can be used to overcome this deficit in the current evidence-base [57, 58].

### Evidence distribution: Species

The top three most-studied species in our map, wild pigs, red deer and roe deer, are the most widely distributed ungulate species in Europe [9]. Wild pigs are invasive alien species throughout much of their range, which covers every continent except Antarctica [30, 59]. This was reflected in our results, which show that more than half (53%, n = 31) of the studies on wild pigs were conducted outside of their native range. They are generalist feeders that reproduce prolifically and are widely regarded as being one of the most destructive invasive species globally [32, 60, 61]. In contrast, almost all of the studies on red and roe deer were conducted within their native ranges (93% and 100% for red and roe deer, respectively). Both species are highly valued for recreational hunting and tourism [8], while overabundant populations can have a negative impact on woodland ecosystems, commercial forestry and agriculture, which makes them priority species for management [8, 9].

The top five countries with the highest frequency of articles were Australia, UK, USA, France and Japan. Feral goats and wild pigs are invasive alien species in Australia and USA and constitute a major threat to native biodiversity [62, 63]. Consequently, there is considerable interest in improving methods of population control and eradication [64–66]. With the exception of Chinese muntjac, all of the ungulate species resident in the UK are also present in France, so it is unsurprising that both countries make a large contribution to the existing evidence-base [9, 53]. The high frequency of studies in Japan is likely driven by the declining popularity of hunting in recent years, which has created a need to explore alternative interventions such as fencing and non-lethal deterrents [67–69].

### Evidence distribution: Interventions

The distribution of evidence across different types of interventions is likely to be influenced by the effectiveness of the intervention, its availability and accessibility to practitioners as well as the range of legal restrictions and cultural views associated with its application. Our results show that a large majority of studies focus on various methods of shooting (78%), including shooting from the ground, from an aerial vehicle and shooting with the assistance of drivers (dogs or human battues). Shooting is popular for a variety of reasons. There is evidence supporting its effectiveness as a tool to mitigate ecological impacts [e.g., 70–72], it is relatively inexpensive [73] and hunting has an important significance in many cultures worldwide [74]. A key advantage of shooting is its specificity, which enables practitioners to target individuals or cohorts within the population (such as senescents, females or diseased individuals), that disproportionally contribute to ecological impacts or may be important for maintaining population health [10, 75]. In contrast, poisoning and capture (trapping and snaring) are less discriminate and so are typically only legally permitted for use on non-native invasive species. Our results show that poisoning and capture were studied for non-native species in 100% (n = 13) and 88% (n = 14) of articles, respectively.

The low proportion (20%) of articles that reported on non-lethal interventions is most likely due to the limited theoretical support for their effectiveness in mitigating ecological impacts. Although the precise relationship between impacts and ungulate population density is complex and context-specific, theoretically there exists a threshold above which species begin to put unsustainable pressure on the environment [33, 34, 76]. Most non-lethal interventions (barriers, diversionary feeding, repellents and deterrents) do not affect population density or reproductive performance and so are more likely to displace the environmental pressure caused by ungulates to other geographic areas, rather than bring about an overall reduction [77, 78]. Immunocontraception may be a viable alternative to lethal interventions and has been successfully developed for more than 85 different wildlife species [79]. However, most of the research on wildlife contraception has focussed on captive populations. There are several factors that currently inhibit the wider use of immunocontraceptive vaccines in free-ranging populations, including the variability of efficacy across species, limited long-term safety testing, the lack of effective delivery systems for elusive and mobile animals and concerns over the potential side-effects on behaviour [79]. Further research is needed to overcome these challenges and achieve general acceptance of immunocontraception as a management tool.

### Evidence distribution: Outcomes

Our results show that most studies focused on population size and space-use outcomes. This is likely because there are established links between these responses and ecological impacts. For example, the relationship between wild ungulate population densities and indices of ecological impact (e.g., forest regeneration) has been investigated in several studies [e.g., 19, 76, 80] and variation in space-use has been linked to the distribution of damage [e.g., 81] as well as the spread of diseases [e.g., 82] and parasites [83]. The types of biological responses examined may also be influenced by data availability. Demographic responses, such as variations in population size, are likely to be observable over much shorter timeframes than changes in physiology or morphology, which require longer periods of population monitoring. Estimating population sizes is relatively straightforward and can be achieved using a range of techniques such as track counts, distance sampling and dung surveys, which require minimal resources. Cull records may also be utilised and are often the only source of population data regularly collected over long timeframes and at regional or national scales [55, 84]. Data on individual health, physiology and morphology are more challenging to collect. Considerable effort is needed to obtain the blood, tissue or whole-organism samples typically required for analyses. Furthermore, accurately measuring indicators of responses, such as stress hormone levels, body condition and the size and shape of anatomical features often requires expertise and specialist equipment that are unavailable to most practitioners [56].

### Recommendations for policy and management

Members of the international scientific community recently advocated for the implementation of an adaptive approach to management of wild ungulate species based on a continuous and systematic process of trial-and-error [14]. They highlight the importance of evaluating the outcomes of management interventions using a set of environmental (e.g., browsing index, vegetation composition, ungulate-vehicle collisions) and population (e.g., body mass, antler quality, reproductive performance) indices. Our results show that, to date, very few studies have utilised population-based metrics beyond estimates of population-size. Therefore, we strongly support the existing call [14] for practitioners to record key information on the health, reproduction and genetic integrity of ungulate populations. We encourage a participatory approach to research, in which managers carrying out adaptive management, become integrated participants in the wider research programme.

As the financial and human resources available to managers are typically limited, it may be sensible firstly to exploit opportunities for broadening the types of data collected from sources already utilised by existing monitoring programmes. For example, cull records could include information on indicators of health, such as body mass, jaw length and antler quality [85]. Blood and tissue samples used for the monitoring of diseases, could also be made available for studies on population genetics and physiology [86, 87]. Data-sharing through collaborative projects, such as the EuroBoar (www.euroboar.org) and EuroDeer (www.eurodeer.org) networks, should be encouraged to facilitate comparative studies of populations under different socio-ecological conditions [56]. The results of alternative strategies are particularly valuable in finding novel solutions to management challenges. For example, a study by [88] proposed shooting in a way that creates a ‘landscape of fear’ to mimic the effects of a natural predator. Critically assessing approaches such as this would facilitate the refinement of existing practices and policies.

### Recommendations for primary research

Researchers should focus on addressing knowledge gaps by conducting studies based on robust experimental designs (such as before-after-control-intervention) that account for different types of bias [89, 90]. Resources should be invested in long-term studies that collect data for 10 or more years, which would provide valuable knowledge on the long-term effects of management and species responses to environmental variation, such as climate and land-use changes [56]. We suggest the following three questions as research priorities: (1) how do ungulate species respond to non-lethal interventions (supplementary feeding, barriers, deterrents and administering contraception)? (2) what are the side-effects of shooting on ungulate (i) morphology, (ii) population genetics, (iii) physiology and (iv) species interactions? and (3) what are the effects of management interventions on sika deer and Chinese muntjac?

Non-lethal interventions provide important alternative methods of mitigating the impacts of ungulates in contexts where lethal interventions are not legally or socially acceptable to use (e.g., urban areas). Understanding species responses to non-lethal interventions is critical for developing more effective techniques and ensuring their long-term safety (e.g., exploring possible side-effects of contraceptives). More research on non-lethal interventions would also assist in identifying the combination of techniques that are most effective at the population-level scale of the landscape or region.

Identifying the side-effects of shooting is important for several reasons. Firstly, shooting is often a non-random process and individuals with certain morphological traits (e.g., large body mass or large antlers) may be preferentially targeted [36, 91]. This can place selection pressures on populations that can cause undesirable life-history changes over shorter time-periods than would be expected from natural selection [36, 92]. Secondly, the relatively slow rate of reproduction exhibited by ungulates puts them at risk of overexploitation [93, 94]. Extensive shooting and anthropogenic barriers (e.g., roads, buildings, fences etc.) can isolate populations, which may increase the rate of inbreeding (i.e., mating among closely related individuals), leading to inbreeding depression [i.e., the decreased fitness of inbred individuals, 93, 95]. Finally, shooting can affect the rate of contact between individuals, which may influence the spread of diseases [96, 97]. There is also evidence to suggest that the social stress of culling activities causes immunosuppression, leading to greater disease expression [98]. There is a need to better understand the full range of side-effects associated with shooting to ensure the long-term viability of ungulate populations and improve management efficiency.

Chinese muntjac and with sika deer, are among the worst invasive non-native species in Europe in terms of risk of causing environmental impacts [99, 100]. In the UK, high densities of Chinese muntjac have been associated with a range of impacts on native species of flora [101], birds [102] and invertebrates [103]. Sika deer present an additional threat to native ungulate species through hybridisation with native red deer populations [e.g., 104–106]. Reliably predicting the responses of Chinese muntjac and sika deer to management interventions is critical in developing effective strategies to reduce population spread. We recommend researchers initially focus on outcomes relating to population growth (e.g., population size, fecundity, survival etc.) and space-use (e.g., distributions, dispersal, movement rates), as they are likely to be the most important factors driving population expansion.

### Recommendations for systematic reviews and meta-analyses

Scholars may look to expand this review by including a broader range of species. Widening the scope of the review to include North American species such as elk (*Cervus elaphus canadensis*), moose (*Alces alces*) and white-tailed deer (*Odocoileus virginianus*) is likely to yield a much greater volume of literature that may provide a more comprehensive overview of the evidence-base. Reviews that include a critical appraisal of the literature should prioritise estimating the relationship between outcomes and environmental factors (e.g., climate or land-use, analysed as ’effect modifiers’ if the data permit a meta-analysis to be carried out). Systematic reviews or meta-analyses are needed to assess the validity of transposing results from one geographic region or ecological context to another. Future reviewers may categorise studies by ecological context and critically evaluate the results to estimate the effects of environmental conditions on species responses.

Our map shows that shooting is the only intervention for which a sufficient volume of evidence currently exists to permit a meaningful systematic review or meta-analysis. Systematic reviews would provide insights on the quality of the literature as well as determining the magnitude, directionality and heterogeneity of effects between different species and ecological contexts (i.e., ’effect modifiers’). Systematic reviews and meta-analyses assessing the relationship between outcomes and variation in shooting practices (e.g., intensity, spatial scale, timing, selectivity) would be particularly valuable for understanding the mechanisms of how shooting works, and what modifiers affect species responses [8, 14].

### Limitations of the search strategy

It is important to consider the limitations of the search strategy when interpreting our study results. Although the searches were comprehensive, finite time and resources prohibited actions, such as combing review papers, forward and backward screening of articles and searching additional databases, which may have yielded a greater number of relevant studies. The searches were also restricted to articles presented in the English language and ’grey’ literature was obtained from UK-based sources only. Efforts to build on this map should focus on expanding the geographic scope of the review by searching for studies from a wider range of sources, ideally through collaborations between multiple reviewers, which provide different institutional accesses and the option of screening articles in a broader range of languages. We also expect that a number of studies exist based on environmental IEC containing information on species responses to management that are not reported in the title or abstract. Such articles would have been excluded at the screening stages of our search strategy in its current form. We recommend that researchers consistently report population-based metrics and, where appropriate, include these details in their title, abstract or keyword list, which will enable future reviewers to more easily access this information.

Additionally, there are more general caveats associated with interpreting the outputs of systematic maps (for further details see CEE guidelines, www.environmentalevidence.org). Firstly, data were extracted to broadly characterise the evidence of linkages between interventions, outcomes and species. The synthesis did not extend to exploring the directionality of effects or estimating average effect sizes, as is typical of systematic reviews or meta-analyses. Secondly, the set of species responses covered in our study was derived from a synthesis of the included articles and is not exhaustive. Assessments of the literature related to other wild ungulate species may identify linkages between interventions and a wider range of outcomes than those reported by the studies in our map. Finally, although study designs give an indication of the robustness of evidence, our map does not provide a critical appraisal of the included articles. A detailed evaluation of how studies mitigate biases and account for heterogeneous effects is needed to more accurately assess the quality of the literature.

## Conclusion

The management of wild ungulate populations should be informed by regular monitoring of both environmental and population-based indicators of ecological change [14, 15]. Our map reveals that the extent of the literature reporting on population-based responses to management is limited. The current lack of primary research constrains our ability to reliably predict the full range of effects an intervention will have on target species, which is critical for developing sustainable, effective and efficient strategies. We encourage researchers and practitioners to monitor a wider range of responses to interventions as an essential part of adaptive population management. New research and the articles identified in this review should be synthesized and, if reliable, utilized as the evidence-base for public policy and management practice decision-making. Although our results suggest that research effort in this field is increasing, the considerable gaps and biases in the current evidence-base need to be addressed before this knowledge can be transferred to real-world applications.

## Supporting information

S1 File ROSES checklist. (XLSX)

S2 File PRISMA checklist. (DOCX)

S3 File Literature searches. (DOCX)

S4 File Database of articles excluded at the full-text level. (XLSX)

S5 File Systematic map database. (XLSX)
