# Supplementary material for: The effects of population management on wild ungulates: A systematic map of evidence for UK species
Source: PLoS One. 2022 Jun 10;17(6):e0267385. doi: 10.1371/journal.pone.0267385 (PMC9187068; doi:10.1371/journal.pone.0267385)
Supplement: S3 File — (DOCX) [file pone.0267385.s003.docx]

**S3. Literature searches**

**Estimating the comprehensiveness of the search**

Each of three reviewers was given the primary question, background, objectives and eligibility criteria for the review and asked to compile a list of 10 articles, including both published peer-reviewed and ‘grey’ literature that they considered suitable for inclusion following a full text analysis. The combined library of 30 articles was used as a test list to assess the efficacy of the search strategy. Only search strings that identified all 30 articles were used for the literature search.

**Searching for articles**

Search terms were tested for sensitivity and specificity in October 2019 using the Clarivate Analytics Web of Science (Core collection) database. Boolean search operators were employed where accepted by a database and wildcards (* and $) were used to detect multiple word endings, for example fenc* would pick up fence, fences, fencing, fenced, etc. and station$ would pick up station or stations. Literature sources that did not accept Boolean search operators were queried with a subset of these terms.

**Publication sources [number of articles identified]**

**Clarivate Analytics Web of Science including: Web of Science Core Collection and BIOSIS Citation Index** 02/03/20

Core [**4065**]

BIOSIS [**4111**]

(muntjac OR "muntiacus reevesi" OR "chinese water deer" OR "hydropotes inermis" OR "roe deer" OR "capreolus capreolus" OR "red deer" OR "cervus elaphus" OR "sika deer" OR "cervus nippon" OR "fallow deer" OR "dama dama" OR "feral goat*" OR "capra aegagrus hircus " OR "wild goat*" OR "feral pig" OR "sus scrofa" OR "feral pigs" OR "feral hog*" OR "feral swine" OR "wild pig" OR "wild pigs" OR "wild hog*" OR "wild boar" OR "feral sheep")

AND

("population control" OR "lethal control" OR hunt* OR cull* OR shoot* OR harvest* OR stalk* OR bait* OR poison* OR trapping OR (inhibit* AND reproduc*) OR immunocontracept* OR contracept* OR "fertility control" OR repel* OR deterrent* OR "diversionary feed*" OR (supplement* AND feed*) OR (supplement* AND food) OR "feed* station$" OR "forest management" OR "landscape structure" OR (manipulat* AND landscape) OR (manipulat* AND habitat) OR fenc*)

**CAB Direct including CAB Abstracts and CABI full text** **(removed "Sus scrofa" term – too many hits for domestic pigs)** 02/03/20

[**4283**]

(muntjac OR "muntiacus reevesi" OR "chinese water deer" OR "hydropotes inermis" OR "roe deer" OR "capreolus capreolus" OR "red deer" OR "cervus elaphus" OR "sika deer" OR "cervus nippon" OR "fallow deer" OR "dama dama" OR "feral goat*" OR "capra aegagrus hircus " OR "wild goat*" OR "feral pig" OR "feral pigs" OR "feral hog*" OR "feral swine" OR "wild pig" OR "wild pigs" OR "wild hog*" OR "wild boar" OR "feral sheep")

AND

("population control" OR "lethal control" OR hunt* OR cull* OR shoot* OR harvest* OR stalk* OR bait* OR poison* OR trapping OR (inhibit* AND reproduc*) OR immunocontracept* OR contracept* OR "fertility control" OR repel* OR deterrent* OR "diversionary feed*" OR (supplement* AND feed*) OR (supplement* AND food) OR "feed* station$" OR "forest management" OR "landscape structure" OR (manipulat* AND landscape) OR (manipulat* AND habitat) OR fenc*)

**Google Scholar (First 100 hits from each intervention term ordered by relevance. Excluded citations and patents. 250-character limit)** 10/03/20

"muntjac" OR "muntiacus reevesi" OR "chinese water deer" OR "roe deer" OR "red deer" OR "sika deer" OR "fallow deer" OR "feral goat*" OR "wild goat*" OR "feral sheep" OR "wild boar" OR "feral pigs" OR "wild pigs" OR "feral hog*"

[AND]

"population control" [**3570**]

hunting [**23200**]

stalking [**14700**]

culling [**14500**]

shooting [**16100**]

poisoning [**20200**]

contracept* [**2930**]

repellent [**6310**]

deterrent [**8250**]

"supplementary feeding" [**2770**]

trapping [**22600**]

fencing [**20700**]

**Animal and Plant Health Agency (APHA)** 17/03/20

https://www.gov.uk/government/organisations/animal-and-plant-health-agency

Research and statistics

[Research only/Statistics (published) only] search using the following terms

Deer [**7**/**0**] – 0 relevant

Goat [**18**/**6**] – 0 relevant

Boar [**5**/**0**] – 0 relevant

Wild Pig [**27**/**1**] – 0 relevant

Feral sheep [**21**/**6**] – 0 relevant

**Department for Environment Food and Rural Affairs (Defra)** 17/03/20

https://www.gov.uk/government/organisations/department-for-environment-food-rural-affairs

Research and statistics

[Research only/Statistics (published) only] search using the following terms

Deer [**2**/**1**] – 0 relevant

Goat [**3**/**3**] – 0 relevant

Boar [**7**/**0**] – 0 relevant

Wild Pig [**20**/**45**] – 0 relevant

Feral sheep [**6**/**45**] – 0 relevant

**Forestry Commission** 17/03/20

https://www.gov.uk/government/organisations/forestry-commission

Research and statistics

[Research only/Statistics (published) only] search using the following terms

Deer [**0**/**0**] – 0 relevant

Goat [**0**/**0**] – 0 relevant

Boar [**0**/**0**] – 0 relevant

Wild Pig [**0**/**0**] – 0 relevant

Feral sheep [**0**/**0**] – 0 relevant

**Forest Research** 17/03/20

https://www.forestresearch.gov.uk/

Publications and research

Deer [**40**] – 0 relevant

Goat [**4**] – 0 relevant

Boar [**17**] – 0 relevant

Pig [**2**] – 0 relevant (excluded 'wild' term – too many irrelevant hits)

Feral sheep [**7**] – 0 relevant

**Natural England** 17/03/20

https://www.gov.uk/government/organisations/natural-england

Research and statistics

[Research only/Statistics (published) only] search using the following terms

Deer [**0**/**0**] – 0 relevant

Goat [**0**/**0**] – 0 relevant

Boar [**0**/**0**] – 0 relevant

Wild Pig [**0**/**0**] – 0 relevant

Feral sheep [**0**/**0**] – 0 relevant

**Natural Resources Wales** 17/03/20

https://naturalresources.wales/evidence-and-data/research-and-reports/?lang=en

Research and Reports

Invasive non-native species reports [**6**] – 0 relevant

Species reports – [**81**] – 0 relevant

**Scottish Natural Heritage** 17/03/20

https://www.nature.scot/

Information Hub

Information Library

Search for: publications and statistics

Deer [**24**] – **4** relevant (including additional material)

Goat [**0**] – 0 relevant

Boar [**0**] – 0 relevant

Wild Pig [**23**] – 0 relevant

Feral sheep [**3**] – 0 relevant

**Game and Wildlife Conservation Trust** 17/03/20

https://www.gwct.org.uk/

Research

Scientific Publications

Deer [**34**] – **1** relevant (also captured from other sources)

Goat [**1**] – 0 relevant

Boar [**8**] – 0 relevant

Wild Pig [**0**] – 0 relevant

Feral sheep [**0**] – 0 relevant

**Department of Agriculture, Environment and Rural Affairs (Northern Ireland)** 17/03/20

Publications

Deer [**6**] – 0 relevant

Goat [**28**] – 0 relevant

Boar [**3**] – 0 relevant

Wild Pig [**74**] – 0 relevant

Feral sheep [**53**] – 0 relevant

**Open Grey** (www.opengrey.eu) 20/03/20

(muntjac OR "muntiacus reevesi" OR "chinese water deer" OR "hydropotes inermis" OR "roe deer" OR "capreolus capreolus" OR "red deer" OR "cervus elaphus" OR "sika deer" OR "cervus nippon" OR "fallow deer" OR "dama dama" OR "feral goat*" OR "capra aegagrus hircus " OR "wild goat*" OR "feral pig" OR "sus scrofa" OR "feral pigs" OR "feral hog*" OR "feral swine" OR "wild pig" OR "wild pigs" OR "wild hog*" OR "wild boar" OR "feral sheep")

AND

("population control" OR "lethal control" OR hunt* OR cull* OR shoot* OR harvest* OR stalk* OR bait* OR poison* OR trapping OR (inhibit* AND reproduc*) OR immunocontracept* OR contracept* OR "fertility control" OR repel* OR deterrent* OR "diversionary feed*" OR (supplement* AND feed*) OR (supplement* AND food) OR "feed* station$" OR "forest management" OR "landscape structure" OR (manipulat* AND landscape) OR (manipulat* AND habitat) OR fenc*)

Screened title and abstract before download as only individual downloads permitted – only immediately downloadable documents were included

Records: **30**

Relevant: **7 (2 available from ETHOS)**

**EThOS** (www.ethos.bl.uk) 06/04/20

Can only use single terms related by Boolean operators up to a maximum of 6 terms

Screened title and abstract before download as only individual downloads permitted – only immediately downloadable documents were included

[all records/**immediate download only**]

deer [287/**192**] – 6 relevant

wild boar [18/**10**] – 2 relevant

feral goat [5/**4**] – 1 relevant

feral sheep [0/0] – 0 relevant

Relevant: **8**

**Article screening**

The lead reviewer (OB) applied the inclusion criteria at the title level to all potentially relevant articles. To check consistency, second (AG) and third (GS) reviewers also assessed a random subset of 200 articles each at the title and abstract level. The level of agreement between reviewers was estimated by calculating Cohen’s kappa coefficient. A value of 0.49 was achieved indicating moderate agreement (1). Articles that passed the title stage, were screened for eligibility by the lead reviewer at the abstract level. The lead reviewer evaluated relevant studies that passed the title and abstract level stages by studying their full text. At this stage, a random subset of 20 articles were assessed by the third reviewer (GS) to check consistency. Articles that were considered ambiguous by one or more reviewers at any stage were marked as equivocal and retained for full text assessment. Equivocal articles were analysed by all reviewers to reach a consensus and the eligibility criteria was modified where necessary. Any reviewer who was the author of a study did not decide on the inclusion of that study and, in cases of uncertainty, the reviewer tended towards inclusion. Data were extracted for articles that met the eligibility requirements at the full text level as described in the “Article screening and data coding” section of the main text.

**References**

1. Landis JR, Koch GG. The Measurement of Observer Agreement for Categorical Data. Biometrics. 1977;33(1):159–74.
